# Structure Identification and Risk Assurance of Unknown Impurities in Pramipexole Oral Drug Formulation

**DOI:** 10.1155/2024/5583526

**Published:** 2024-02-13

**Authors:** Raymond R. Tjandrawinata, Antonius H. Cahyana, Ajeng O. Nugroho, Indra K. Adi, Joseph S. R. Talpaneni

**Affiliations:** ^1^Atma Jaya Catholic University of Indonesia, South Jakarta 12930, Indonesia; ^2^Department of Chemistry, Faculty of Mathematics and Natural Science, University of Indonesia, Central Jakarta 10430, Indonesia; ^3^Dexa Development Centre, PT Dexa Medica, Industri Selatan V Blok PP-7, Jababeka Industrial Estate, Cikarang 17550, Indonesia

## Abstract

Impurities compounds in any pharmaceutical product or drug substance are inevitable from a chemistry point of view. The quality and safety of a pharmaceutical product are also significantly affected by these impurities content; therefore, impurities need to be identified and characterized through the use of appropriate analytical methods. Pramipexole is a nonergot dopamine agonist used to treat various Parkinson's disease symptoms. Two unknown impurities were detected from a pramipexole dihydrochloride solid dosage form. These impurities were identified and characterized using ultra-performance liquid chromatography coupled with high-resolution mass spectroscopy (UPLC-HRMS). These impurities were found to be enriched when mannitol existed in the formulation. The structure and mechanism involved in the existence of the impurities were proposed. Furthermore, observation of the binding affinity potential risk of these impurities to the pramipexole receptor has also been demonstrated through molecular docking and molecular dynamics simulation study. The binding energy result showed that pramipexole interaction with dopamine receptors D2 and D3 was higher than pramipexole mannose adduct and pramipexole ribose adduct.

## 1. Introduction

**Figure 1:**
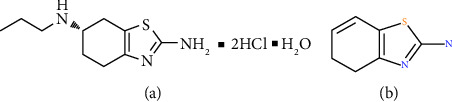
(a) Molecular structure of pramipexole dihydrochloride monohydrate [1] and (b) its fragmentation.

Pramipexole dihydrochloride monohydrate (Figure 1) is an active substance that acts as an anti-Parkinson. Pramipexole binds selectively to the D2 dopamine receptor subfamilies and has more affinity for the D3 dopamine receptor. It is well-established as a treatment option for motor symptoms at all stages of Parkinson's disease (PD). Also, this drug is effective in the treatment of idiopathic and secondary restless legs syndrome (RLS) and in treatment-resistant patients as well [1–4].

The therapeutically active product comprises active pharmaceutical ingredients (APIs) and excipients. The API is responsible for producing pharmacological effects after absorption in systemic flow in the living body. But in some circumstances, the active constituent or excipients could not be 100% pure and may contain other components that may arise in the medicinal product from different sources, i.e., from synthesis, an excipient, residual solvent, or degradation product. These unwanted components other than API and excipients are known as impurities. Impurity is the product or substance formed in the synthesis, including intermediate or the side product of intermediate that formed during the side reaction or unwanted chemical reaction [5–8].

Many impurities in a drug product can be obtained from excipients used to formulate a drug substance. The excipient can sometimes interact with the main ingredient to produce an undesirable product [9]. The interaction product for all practical purposes is considered an impurity (or impurities) [10–12]. Excipients are known to facilitate administration and modulate the release of the active component. They can also stabilize it against degradation from the environment. Most excipients have no direct pharmacological action, but they can impart useful properties to the formulation. However, they can also give rise to inadvertent and/or unintended effects, such as increased degradation of the drug. Physical and chemical interactions between drugs and excipients can affect the chemical nature, the stability and bioavailability of drug products, and consequently, their therapeutic efficacy and safety [13–16].

Identification of pharmaceutical impurities is a critical analytical activity in the drug development process whose goal is to elucidate the chemical structure of unknown pharmaceutical impurities fully present in either drug substances or drug products above a particular threshold [17]. Impurity profiling is a systemic process to identify the unknown impurity and isolate the impurity to elucidate the structure. It is an important approach designed for identifying and quantifying the impurities existing in the medicinal substance [7, 18, 19].

Hyphenated liquid chromatography methods, especially those coupled with a mass spectrometer as the detector, have been widely used to separate and identify possible existing impurity from a drug substance and/or drug formulation. Furthermore, the use of high-resolution mass spectrometry (HRMS), employing quadrupole time-of-flight (Q-TOF) detector, is gaining more popularity in profiling pharmaceutical impurity since it is capable of high-resolution mass detection (up to submilli-Dalton level) which would then provide high accuracy for structure and reaction mechanism prediction. Electrospray ionization in the positive mode is the preferred method in pharmaceutical impurity analysis, mainly due to its ability to detect thermally labile, nonvolatile, and polar compounds [20–30].

## 2. Materials and Methods

### 2.1. Identification of Impurities

Pramipexole dihydrochloride monohydrate (PRM) batch number PH008116 (%assay based on its manufacturer CoA was 100.1%) was procured from Hetero Drugs Ltd (India) and used without further purification. Pramipexole dihydrochloride tablets (PR-FP) were obtained from the development of the Formulation Development Division of PT Dexa Medica's Research and Development Department. Mannitol (spray-dried grade) batch number EF91G (%assay based on its manufacturer CoA was 99.0%) was procured from Roquette (France) and used without further purification. Ammonium formate (MS grade) and formic acid (MS grade) were procured from Sigma-Aldrich, Singapore. Acetonitrile (MS grade) was procured from Merck Millipore, Singapore.

Thermal degradation was carried out inside a hot-air oven (Daihan ThermoStable OV-70) capable of controlling temperature within the range of ±5°C. Informal stability studies were carried out using (Climatic Chamber KBF 720 and Newtronic Walk-In Chamber). The informal stability study (ISS) samples were analyzed on a Waters high-performance liquid chromatography (HPLC) system (Milford, MA, USA) consisting of a quaternary pump, PDA detector, and autosampler. The data were acquired and processed in Empower 3 software. The chromatographic separations of the HPLC system were achieved on the Zorbax RX C8 column (250 × 4.6 mm, 5 *µ*m). Mass analysis was carried out using a Waters UPLC system (Milford, MA, USA) equipped with a quaternary pump, PDA detector, and autosampler and coupled with a Xevo G2-XS Qtof mass spectrometer (Waters, USA) operated in the electrospray ionization (ESI) mode. The chromatographic separations of the UPLC system were achieved on the Acquity UPLC BEH C8 column (100 × 2.1 mm, 1.7 *µ*m). The data were acquired and processed in MassLynx software. Molecular docking was performed using AutoDock Vina software, while molecular dynamics simulations were processed using YASARA software.

### 2.2. LC-UV Analysis of Informal Stability Samples [14, 31]

A PR-FP batch sample was subjected to an informal stability study, in which the drug products were exposed to conditions of 30°C/75% RH and 40°C/75% RH for up to 8 weeks. The sample was analyzed using an HPLC system consisting of a Zorbax RX C8 column (250 × 4.5 mm, 5 *µ*m, 30°C column temperature) using 260 nm as the detection wavelength. The mobile phase consisted of MP A (0.2% triethylamine in pH 6.0 ammonium formate buffer: acetonitrile, 98 : 2 v/v) and MP B (acetonitrile), run on gradient elution as shown in Table 1. The mobile phase flow rate was 1.0 mL/minute, and each sample was injected at 200 *µ*L of volume (the analysis was performed with a higher injection loop). For each analysis, 10 tablets of sample were sampled and dissolved with pH 6.0 ammonium formate buffer as diluent up to 25 mL.

### 2.3. LC-HRMS Analysis of Simulation Samples

Simulation samples using a synthetic mixture of PRM and mannitol were made with proportional composition to the formula of PR-FP. Application of heat at a temperature of 105°C for 6 hours is done to intentionally grow the unknown impurities. The sample would be analyzed using a UPLC-HRMS system consisting of an Acquity UPLC BEH C8 column (100 × 2.1 mm, 1.7 *µ*m, 30°C column temperature). The mobile phase consisted of MP A (5.0 mM pH 6.0 ammonium formate buffer) and MP B (acetonitrile), run on gradient elution mode as per shown in Table 2. The mobile phase flow rate was 0.3 mL/minute, and each sample was injected at 5 *µ*L of volume using MP A as diluent during preparation. For each analysis, an equivalent amount of 10 tablets (mixture of 1.25 mg of PRM and 820 mg of mannitol) were sampled and dissolved with diluent to 10 mL and then further diluted with diluent to reach 50 *µ*g/mL concentration of PRM. Simulation samples were subjected to an MS system in positive electrospray ionization (ESI+) in the mass range of 50–800 Da. High-purity nitrogen was used as the nebulizer and collision gas. The operating condition for HRMS was optimized as follows: capillary voltage 2.5 kV; cone voltage 15 V; collision energy 15 V; desolvation temperature 450°C; and desolvation gas flow 800 L/hour.

### 2.4. In Silico Study

#### 2.4.1. Preparation of Proteins and Ligands

3D structure of the protein and ligand (pramipexole, pramipexole mannose adduct, and pramipexole ribose adduct) used in the experiment was converted using OpenBabel from its SMILES (Simplified Molecular Input Line Entry System) data obtained from website https://www.synzeal.com/en [32]. Their conformers were then optimized using obconformer [33]. ORCA software was employed for further geometry optimization using the B3LYP quantum theory and Def2-SVP DFT level [34, 35]. They were then converted to pdb using OpenBabel and further converted into pdbqt using the prepare_ligand module from ADFR. The protein structures of the dopamine D2 receptor (PDB ID:7JVR) and dopamine D3 receptor (PDB ID: 7CMU) were retrieved from the Protein Data Bank website (https://www.rcsb.org). Ligands, metal, water, and other hetero atoms were removed from protein using SPORES [36]. Protein was then converted into pdbqt using the prepare_protein module from ADFR [37]. The binding site definition (coordinate and size) was obtained using PLANTS [38].

#### 2.4.2. Molecular Docking

Molecular docking studies of pramipexole, pramipexole mannose adduct, and pramipexole ribose adduct on both dopamine D2 and D3 receptors were performed using AutoDock Vina. Ten poses for each ligand were generated and the best ligand pose (Pose 1) was complexed with D2 and D3 receptors using Pymol [39, 40]. The protein-ligand complexes were then further simulated using Molecular Dynamic Simulation.

#### 2.4.3. Molecular Dynamics Simulation

Molecular dynamics simulation was performed using YASARA Structure Software to evaluate structural dynamics, conformational behavior, and stability of the protein and protein-ligand complexes. Protein-ligand complexes were minimized using em_clean macro from YASARA and Amber14 Force Field. Membrane lipid composed of phosphatidyl-ethanolamine (PEA) was then added to the system using md_runmembrane macro as it is considered the most stable membrane lipid. Molecular dynamics simulation was run for a 50 ns timescale in 0.9% sodium chloride as the condition that replicates physiological conditions. The simulation was performed using pH 7.4 and temperature 298 K as the default setting.

## 3. Results and Discussion

### 3.1. Informal Stability and Incompatibility Study

**Figure 2:**
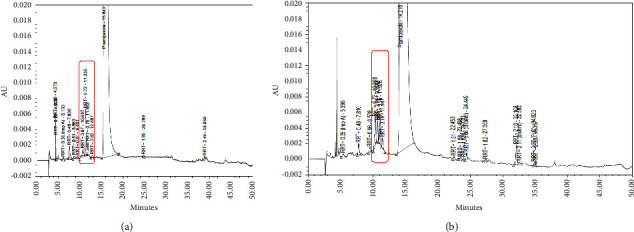
HPLC-UV trace of injected samples. (a) HPLC-UV trace of PR-FP sample and (b) PRM-mannitol mixture sample.

An in-house developed HPLC method was used to separate and quantify impurities of PR-FP in a single run. Upon analysis of ISS samples, two unknown impurities were detected as two unresolved peaks at relative retention time (RRT) ∼0.7 (see Figure 2(a)). The number of unknown impurities was also shown to increase during the study and at increasing temperature and was also found to exceed the internal specification limit of not more than 0.44% w/w. The detailed ISS result is given in Table 3.

Since the unknown impurities were found to be growing during ISS, it is necessary to identify the structure and origin of the unknown impurities to refine and control the quality of the drug product. The formula of pramipexole dihydrochloride tablets (PR-FP) consists of pramipexole as active pharmaceutical ingredients and several inactive pharmaceutical ingredients (excipients) with detailed composition as shown in Table 4.

Based on the incompatibility study experiment, the same peak pattern was also observed in the sample consisting of a mixture of PRM and mannitol (see Figure 2(b)), and the value of the impurities was significantly increased after exposed by heat (see Table 5). Mannitol used in this research contains impurities, according to its CoA manufacturer data, in the form of reducing sugar (reported value < 0.1%), d-sorbitol (reported value 0.9%), and a total of impurity B and C (reported value 0.05%).

### 3.2. Identification of Impurities by HRMS

**Figure 3:**
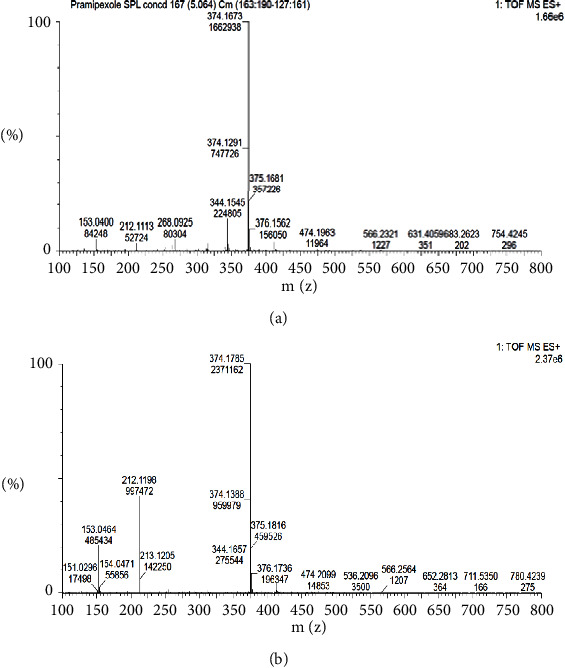
Mass scan of samples. (a) PR-FP sample and (b) PRM-mannitol mixture.

In the UPLC system, the unknown impurities were detected as one single peak at RRT ∼0.89. The mass scan of peak RRT∼0.89 showed four distinct masses with m/z of 153.0464, 212.1198, 344.1657 (Imp 1), and 374.1785 (Imp 2). This result shows that the target impurity may consist of more than one compound. Some identified masses (m/z of 212.1198 and 153.0464) were omitted from further interpretation as both belong to pramipexole and its fragment, respectively (Figure 1(b)). The pattern of mass scan observed in both PR-FP and the mixture of PRM-mannitol (Figures 3(a) and 3(b), respectively) shows that they share similar masses which leads to the possibility of the targeted impurities coming as a result of interaction between both molecules.

### 3.3. Formation of Impurities, Simulation, and Structure Validation Studies

The structure of pramipexole as the active ingredient shows that it has primary amine. The amine functional group can act as nucleophiles in relation to the unshared electron pair it has. The formula of the solid dosage form of pramipexole consists of mannitol as one of its excipients. Mannitol is a sugar alcohol derived from a sugar by reduction and is a commonly used, nonhygroscopic, and chemically stable excipient, with good flow properties and high compressibility. As such, it is suitable to be used with water-sensitive APIs. It is primarily used as a diluent (10–90%) in tablet formulations. There have, however, been some reports of adverse effects of mannitol on the stability of drugs [20, 41, 42].

**Figure 4:**
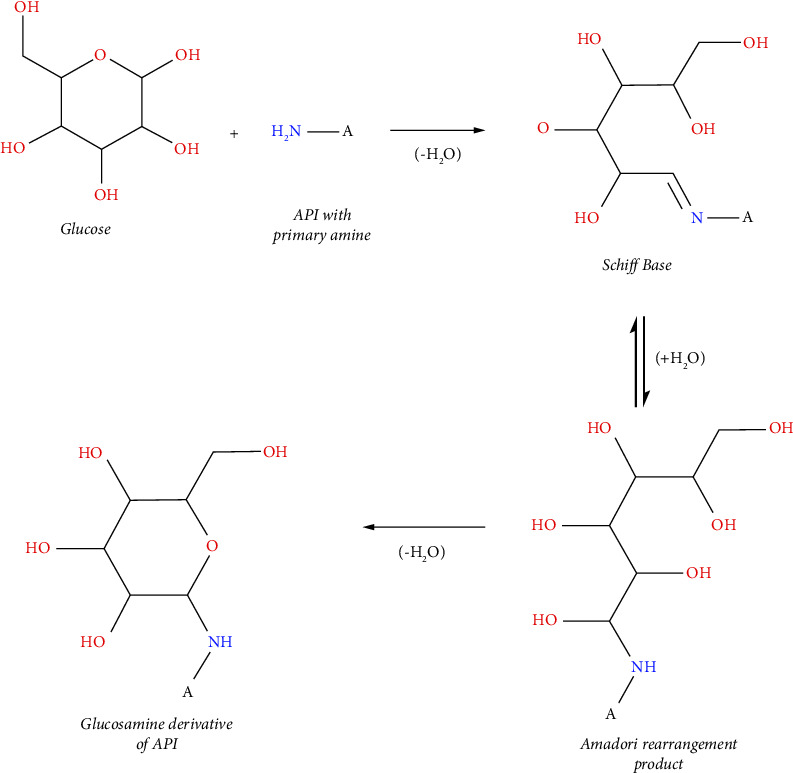
Maillard reaction between API with primary amine and a reducing sugar (i.e., glucose).

**Figure 5:**
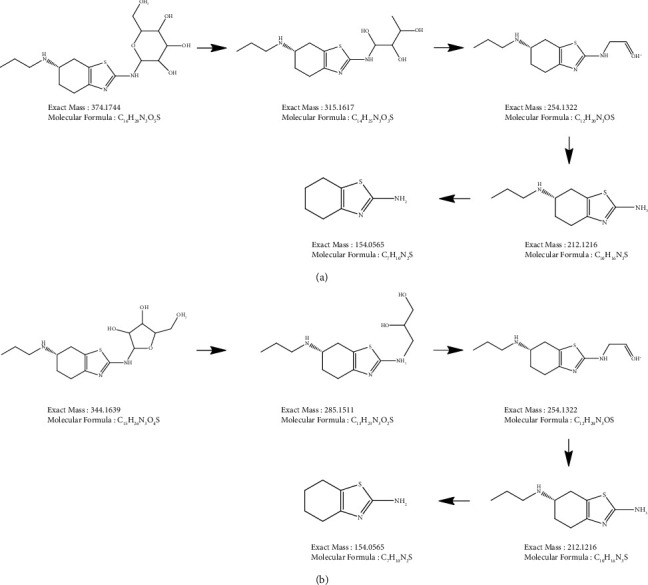
Proposed structure of unknown impurities in pramipexole oral drug formulation. (a) Imp 2 (m/z 374) and its fragmentation scheme and (b) Imp 3 (m/z 344) and its fragmentation scheme.

Maillard reaction (Figure 4) is a common incompatibility mechanism between APIs with primary amine group with reducing sugar from its excipient in drug formulation [43]. The reaction would result in glucosamine-derivate of API, which is promoted at high temperatures. In this case, the reducing sugar meant before may be contained inside the mannitol substance itself as an impurity such as fructose, glucose, or mannose. Those reducing sugars are known to be involved in the production of mannitol, whether as the initial precursor or as a byproduct. The illustration presented in Figure 5(a) shows that impurity 2 may be the result of the Maillard reaction between pramipexole and reducing sugar impurity from mannitol, specifically with an initial MW of 180 Da. Following the thought process of impurity 2's explanation, impurity 1 may be the result of the Maillard reaction between pramipexole and other reducing sugar impurities, specifically with a shorter C chain (furanose group) (Figure 5(b)) [44–46].

To confirm this hypothesis, a study was carried out to simulate the manufacturing process of pramipexole dihydrochloride monohydrate tablets with the introduction of thermal stress conditions to ramp up the formation of the sugar-adduct impurities. From the ESI (+) trace and mass scan, it is shown that the sugar-adduct impurities appeared significantly from the wet-granulation and heated samples compared to their control counterpart (without the addition of water and heating, respectively). Elemental composition analysis using MassLynx software also confirmed that the obtained impurities from the simulation study samples matched the chemical structure of the proposed impurities. The observed phenomena thus supported the given proposal of impurities formation through the Maillard reaction between pramipexole dihydrochloride monohydrate and mannitol (in the form of its reducing sugar impurities) in the presence of water and heat.

**Figure 6:**
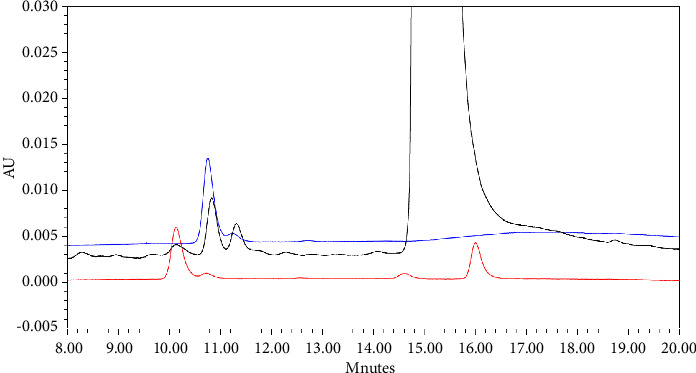
Injection of sample solution and standard of targeted impurities (black line: sample solution; blue line: standard solution of pramipexole mannose adduct; and red line: standard solution of pramipexole ribose adduct).

A validation study experiment was employed to strengthen the proposal structure of the targeted unknown impurities in pramipexole oral drug formulation. The primary standard of the targeted impurities molecule, synthesized by Toronto Research Chemicals, was injected into the same HPLC-UV system used for the detection of unknown impurities in the earlier step of the experiment [47]. As shown in Figure 6, the standard of both pramipexole mannose adduct and pramipexole ribose adduct was eluted in relative retention time around 0.7, the area where the targeted unknown impurities also eluted previously.

### 3.4. Molecular Docking and Molecular Dynamics Simulation

**Figure 7:**
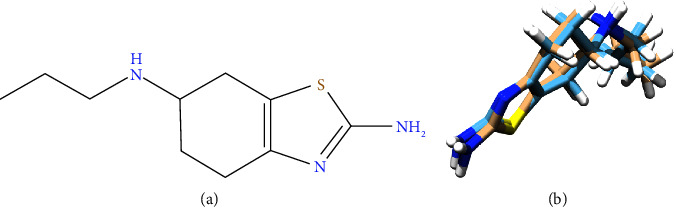
(a) 2D structure of pramipexole and (b) 3D structure of pramipexole after optimization using ORCA.

**Figure 8:**
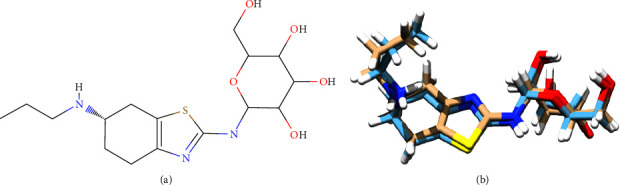
(a) 2D structure of pramipexole mannose adduct and (b) 3D structure of pramipexole mannose adduct after optimization using ORCA.

**Figure 9:**
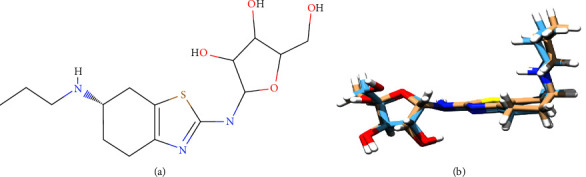
(a) 2D structure of pramipexole ribose adduct and (b) 3D structure of pramipexole ribose adduct after optimization using ORCA.

Since the impurities of pramipexole oral drug formulation have been identified, we have to know whether they have similar activity with pramipexole. Therefore, molecular docking and molecular dynamics were conducted towards an optimized 3D structure of the pramipexole and its impurities (see Figures 7–9) at pramipexole's targeted receptors [48, 49].

Pramipexole is known as a dopamine receptor agonist, especially the dopamine D2 receptor subfamily. The dopamine D2 receptor subfamily consists of several receptor subtypes such as D2, D3, and D4. Pramipexole is mainly active at D2 and D3 receptors and its binding affinity for D3 receptors is higher than D2 receptors. Hence, in this molecular docking study, dopamine D2 and D3 receptors were used as targeted receptors [50].

Molecular docking aims to predict the potentiality of the molecules to engage to the binding site of the targeted protein on its stationary conditions. After completing the molecular docking study using AutoDock Vina software, 10 poses for each ligand were generated. The best ligand pose (pose 1) was then complexed with D2 and D3 receptors and further simulated using molecular dynamic simulation.

Molecular dynamics simulation of the best pose from ligand binding of three targeted molecules was employed at physiological conditions and with the addition of a membrane. The addition of a membrane is important because the dopamine receptors family is known to be one of the subgroup variants of G protein-coupled receptors (GPCRs) which represent the most important drug targets. GPCRs itself are known to be embedded in a cell's plasma membrane [51]. The structure, dynamics, and function of the receptors are influenced significantly by the membrane environment. Hence in this study during the molecular dynamics simulation, we consider inserting a membrane into the protein structure. The root mean standard deviation (RMSD) is the measure of the deviation of the protein backbone from its initial structure and conformation to its final conformation. The deviation produced throughout the simulation determines the stability of the protein. The ligands binding to the protein are considered stable if the deviation of the RMSD of the backbone atoms of the protein in the last 5 ns of the molecular dynamics simulations is less than 2 angstroms [20].

**Figure 10:**
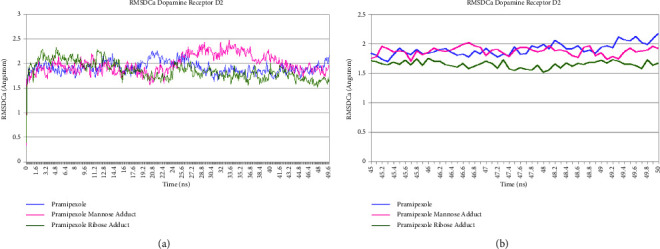
RMSDCa graphic of pramipexole, pramipexole mannose adduct, and pramipexole ribose adduct in dopamine receptor D2. (a) 50 ns trajectories and (b) in 5 ns trajectories.

**Figure 11:**
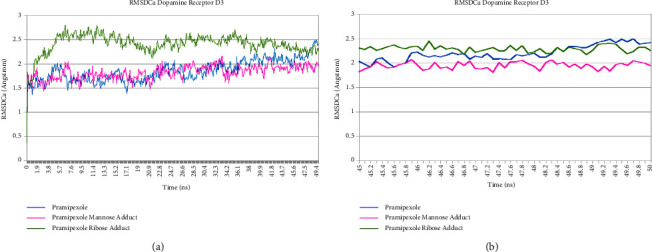
RMSDCa graphic of pramipexole, pramipexole mannose adduct, and pramipexole ribose adduct in dopamine receptor D3. (a) 50 ns trajectories and (b) 5 ns trajectories.

**Figure 12:**
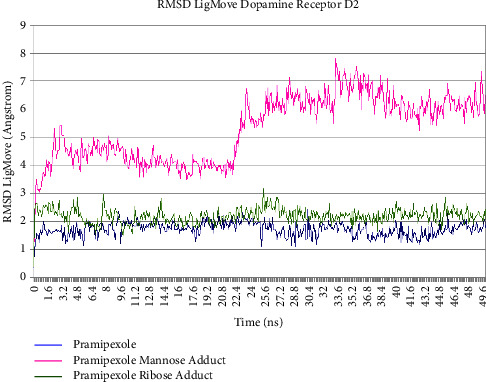
RMSD LigMove of pramipexole and its identified impurities towards dopamine receptor D2.

**Figure 13:**
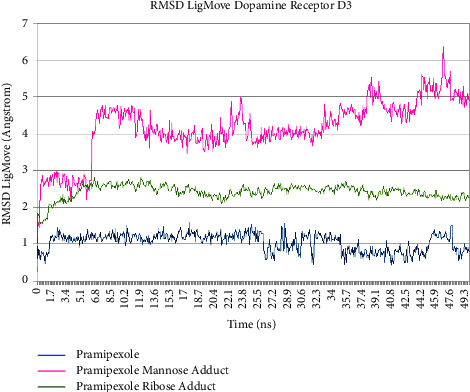
RMSD LigMove of pramipexole and its identified impurities towards dopamine receptor D3.

Pramipexole, pramipexole mannose adduct, and pramipexole ribose adduct showed no significant fluctuation of the RMSDCa value along the last 5 ns trajectory during molecular dynamics simulation time, as shown in Figures 10 and 11. The tabulation of the calculated deviation of the RMSD value of the studied complexes with respect to the C*α* atom throughout molecular dynamics simulation time was 0.091 Å, 0.125 Å, and 0.068 Å for pramipexole, pramipexole mannose adduct, and pramipexole ribose adduct in dopamine receptor D2, respectively. Meanwhile, in dopamine receptor D3, the calculated deviation of the RMSD value was 0.182 Å, 0.129 Å, and 0.053 Å for pramipexole, pramipexole mannose adduct, and pramipexole ribose adduct, respectively. Both data show the stability of the ligand-protein complex of pramipexole, pramipexole mannose adduct, and pramipexole ribose adduct towards dopamine receptors D2 and D3 as it shows less than 2 angstroms of the deviation RMSD value. After molecular dynamics simulation, aside from the RMSDCa value, observation of ligand movement through its RMSD value can also give valuable input on the determination potentiality of the identified impurities to interact with the pramipexole receptor.  Figure 12 (dopamine D2) and Figure 13 (dopamine D3) are the graphic of the RMSD LigMove value of pramipexole, pramipexole mannose adduct, and pramipexole ribose adduct. These graphics suggested that the pramipexole identified impurity, which was pramipexole mannose adduct, tends to leave the dopamine receptors D2 and D3 protein interaction site compared to pramipexole itself and the other identified impurity, which was pramipexole ribose adduct.

Finally, observation of targeted molecules' binding energy to the receptor can be done after molecular dynamics simulation. Binding energy is the total of all the nonbonded interactions. Binding energy was calculated for the last 5 ns of the MD trajectory and tabulated in Table 6. The binding energy towards dopamine receptor D2 for pramipexole, pramipexole mannose adduct, and pramipexole ribose adduct was 83.021 kJ/mol, −4.605 kJ/mol, and −180.197 kJ/mol, respectively. Whereas to dopamine receptor D3, the binding energy was 149.219 kJ/mol, −53.827 kJ/mol, and −138.741 kJ/mol for pramipexole, pramipexole mannose adduct, and pramipexole ribose adduct, respectively. By using YASARA software, more positive energies indicate better binding; therefore, the result showed that pramipexole had a higher binding affinity towards dopamine receptors D2 and D3 compared to its identified impurities, pramipexole mannose adduct and pramipexole ribose adduct.

Pramipexole, as the active pharmaceutical ingredients in the tested solid drug formulation, can stimulate dopamine receptors and further allow patients to control their movement and mitigate the symptoms of Parkinson's disease. These research data suggest that both identified pramipexole impurities would not bind tightly to the binding site of the receptor and hence did not alter the selective and specific binding of pramipexole to its receptor to further produce their desired pharmacological effect.

## 4. Conclusion

The unknown impurities detected from the UV trace of the pramipexole tablet analysis have been profiled using UPLC-HRMS. Based on the acquired m/z data, fragmentation pattern, and knowledge of the formulation process involved, it is suspected that the unknown impurities were enriched due to the addition of mannitol. An incompatibility study was conducted to simulate the reaction between pramipexole dihydrochloride monohydrate with mannitol in heated conditions. The obtained impurities product from the incompatibility study matched the origin analyzed data of the UV-trace. Thus, it has been proposed that the unknown impurities are a product(s) of pramipexole dihydrochloride monohydrate and mannitol, following the Maillard reaction between pramipexole and reducing sugar impurities of mannitol. From all the docking and molecular dynamics simulation data, we can conclude that both identified impurities of pramipexole, which were pramipexole mannose adduct and pramipexole ribose adduct, would unlikely to interact with the active binding site of pramipexole in dopamine receptors D2 and D3 protein compare to pramipexole itself.

## Figures and Tables

**Table 1:** HPLC-UV gradient programme for analysis of informal stability samples.

Time (minutes)	Mobile phase A (%)	Mobile phase B (%)
0	100	0
10	97	3
25	80	20
35	65	35
45	60	40
46	100	0
50	100	0

**Table 2:** UPLC-HRMS gradient programme for analysis of simulation samples.

Time (minutes)	Mobile phase A (%)	Mobile phase B (%)
0	100	0
2.78	97	3
6.95	80	20
9.73	65	35
12.5	60	40
12.78	100	0
14.00	100	0

**Table 3:** Found quantity of largest unknown impurities from informal stability sample analysis.

Temperature (°C)	Found quantity at RRT around 0.7 (%w/w)			
	0^th^ week	2^nd^ week	4^th^ week	8^th^ week
30	0.29	—	1.64	2.0
40		1.32	2.32	—

**Table 4:** Formula composition of PR-FP.

Ingredients	Function	mg/tab	%
Pramipexole dihydrochloride monohydrate	Active pharmaceutical ingredients	0.25	0.15
Mannitol	Excipients	164.5	95.6
Low-substituted hydroxypropyl cellulose (LHPC-LH11)	Excipients	3.4	2
Magnesium stearate	Excipients	3.4	2
Colloidal anhydrous silica	Excipients	0.17	0.1

**Table 5:** Incompatibility study of pramipexole and its excipients.

Sample ID	Condition	Unknown imp (RRT∼0.7)
Pramipexole	W2 40°C	ND
	W4 40°C	ND

Mixture of pramipexole + mannitol	W2 40°C	0.04
	W4 40°C	0.38

Mixture of pramipexole + low hydroxypropyl cellulose	W2 40°C	ND
	W4 40°C	ND

Mixture of pramipexole + colloidal anhydrous silica	W2 40°C	ND
	W4 40°C	ND

Mixture of pramipexole + Mg stearate	W2 40°C	ND
	W4 40°C	ND

**Table 6:** Binding energy of pramipexole, pramipexole mannose adduct, and pramipexole ribose adduct towards dopamine receptor D2 and dopamine receptor D3.

Molecule	Binding energy (kJ/mol)	
	Towards dopamine receptor D2	Towards dopamine receptor D3
Pramipexole	83.021	149.219
Pramipexole mannose adduct	−4.605	−53.827
Pramipexole ribose adduct	−180.197	−137.741

## Data Availability

The data used to support the findings of this study are included within the article.
